# Evaluation of a Brief Intervention to Improve the Nursing Care of Young Children in a High HIV and AIDS Setting

**DOI:** 10.1155/2012/647182

**Published:** 2012-03-15

**Authors:** Linda M. Richter, Tamsen J. Rochat, Celia Hsiao, Thembelihle H. Zuma

**Affiliations:** ^1^Human Sciences Research Council and University of the Witwatersrand, South Africa, 750 Francois Road, Durban 4001, South Africa; ^2^Africa Centre for Health and Population Studies, University of KwaZulu Natal, P.O. Box 198, Mtubatuba 3935, South Africa; ^3^Department of Psychology, University of Western Ontario, Westminster Hall, 361 Windermere Road, London, Ontario, Canada N6A 3K7

## Abstract

The HIV epidemic in South Africa is putting great strain on health services, including the inpatient care of young children. Caregivers and young children (107 pairs) and 17 nurses participated in an intervention to improve the care of young children in hospital in a high HIV and AIDS setting. The intervention addressed caregiver expectations about admission and treatment, responsive feeding, coping with infant pain and distress, assistance with medical procedures, and preparation for discharge and home care. Following a preparatory and piloting phase, measures of nurse burnout, caregiver physical and emotional well-being, and caregiver-child interaction were made before and after intervention. No changes were found between before and after intervention on assessments of caregiver wellbeing. However, mothers in the postintervention phase rated nurses as more supportive; mother-child interaction during feeding was more relaxed and engaged, and babies were less socially withdrawn. While the intervention proved useful in improving certain outcomes for children and their caregivers, it did not address challenging hospital and ward administration or support needed by caregivers at home following discharge. To address the latter need, the intervention has been extended into the community through home-based palliative care and support.

## 1. Introduction

 HIV and AIDS have detrimental effects on children's lives in South Africa, a country that has the largest number of people living with AIDS. HIV/AIDS is the leading cause of death among children under the age of 5 [1], with approximately 330.000 children presently living with HIV/AIDS [2]. In KwaZulu-Natal, the worst affected province in the country, and where the current study was undertaken, approximately 93,000 children under the age of 15 were living with HIV in 2006 [3]. In 2008, HIV prevalence among antenatal women was estimated at 29%, while in KwaZulu-Natal, the provincial site of the study, it was as high as 39 percent [4]. Despite the dire need, it was estimated that in 2005 only 10 percent of children in KwaZulu-Natal in need of antiretroviral treatment received it [3]. Without access to such treatment, the rapidly debilitating health of children living with HIV leads to multiple hospital admissions before they succumb to illness and die.

 These epidemic conditions have profound implications for health care systems, including services for children. More than 60% of pediatric wards in public hospitals are occupied by children admitted with AIDS-related illnesses with respiratory, gastrointestinal, and neurological symptoms [5–7]. Enormous pressure is placed on nurses in pediatric wards in public hospitals as a result of the increased responsibility for caring of higher numbers of acutely and terminally ill children, and the emotional burden of witnessing the deterioration and death of young children in their care. This is compounded by nurse training, skills, and support that are not always equivalent to the demands of care. As a means of coping, nursing staff often become emotionally withdrawn, less compassionate, and burntout [8]. Such detached stance cannot only directly compromise the quality of child care, but can often lead to depletion in nurses' resources to communicate with caregivers about their children's conditions and to support caregivers' participation in their children's care. Without the appropriate support and guidance on how to deal with their child's illness and hospitalization, caregivers often feel frustrated, helpless, disempowered [9], and potentially unresponsive to their children's needs. Indeed, many researchers have highlighted the struggle that healthcare workers face in involving parents' participation in delivering care to their children while in hospital [10, 11]. Under these strained conditions, children's medical, nutritional as well as their social and emotional needs may be neglected. Children move between states of fretful sleep, distressed crying, and withdrawn immobility [8]; as a result of being understimulated in interaction with unresponsive caregivers, children may become socially withdrawn, feed poorly, and recover unevenly.

 Taken together, there is great impetus to develop, implement, and evaluate cost-effective interventions that aim to mitigate the care burden on nursing staff, promote supportive nurse-caregiver relationships and communication to facilitate a partnership in delivering nursing care to children, and ultimately improve the care of children living with HIV in resource-poor hospitals. The aim of this study was to evaluate the effectiveness of such an intervention. The rationale for and details of the intervention can be found in Govender et al. [12] and in Richter et al. [8]; here we provide only a brief overview to serve as a context for the evaluation.

 The intervention was founded upon the premise that increases in nurses' insight, understanding, knowledge, and skills in dealing with acutely ill children through training and support [13], and the promotion of caregivers' involvement in their children's care [14] can significantly benefit the quality of care delivered to hospitalized children. The intervention package included five, short educational videos created to demonstrate to nursing staff and caregivers solutions to difficulties in caring for hospitalized children affected by HIV/AIDS from extensive naturalistic video recordings made of daily care in the ward. By witnessing nurses, mothers, and children overcoming problems in ordinary ways that could be implemented by everyone, we aimed to build cycles of empathy amongst nurses, caregivers, and children. The videos were geared toward training and encouraging nursing staff to communicate with and teach caregivers simple and useful techniques that could be employed in five specific intervention areas: (1) preparing caregivers for hospital admission by communicating to them what would be expected from them. With greater confidence and better informed expectations, caregivers can provide a social reference that helps their children overcome undue fear and anxiety; (2) teaching caregivers simple techniques to encourage actively and responsively feeding their children in order to ensure sufficient nutrition; (3) showing caregivers various methods on how to cope with and comfort children's crying and distress; (4) involving caregivers in ward routines and medical procedures such as washing, blood draws, administering medication and setting up drips; (5) preparing caregivers for discharge by helping to increase caregiver confidence in their capacities to provide good-quality care for their children at home and by reassuring caregivers that the nursing staff is available should they have any questions and concerns before they leave.

 The focus of the current paper is to describe our efforts to evaluate the impact of the intervention on: caregivers' overall psychological well-being and their perceptions of support received from nurses during their children's hospitalization; the quality of interactions between caregiver and child during various exchanges including feeding sessions as well as children's social withdrawal behavior. We also provide qualitative descriptions of the effects of the intervention on the overall ward atmosphere and nurses' burnout and compassion fatigue, as nurses' well-being may potentially impinge on the quality of care delivered to children. Details from the nurse data have been reported elsewhere [15], and the focus of this paper is on the impact of the intervention on caregivers and children.

## 2. Materials and Method

### 2.1. Participants

The participants in this intervention evaluation were nurses, caregivers, and their children recruited into a support programme aimed to reduce the burden on nurses and caregivers in order to improve the care environment for young children admitted to the paediatric ward of an overburdened public hospital in Durban, KwaZulu-Natal, South Africa. The focus of this paper will be on data recorded for caregivers and their children, while information concerning the nursing staff serves as a background.

#### 2.1.1. Nurses

There was always a minimum of 15 nurses on duty in the ward. In order to standardize exposure and intervention dose effects, research staff ensured that all nurses working in the ward during the intervention period had received training in delivering the intervention videos, and that they introduced the intervention to caregivers as part of routine practice in the ward; however, due to high levels of staff rotations in the hospital, many nurses who participated in the preintervention phase of the study were not able to participate in the postintervention phase. As a result, 18 nurses participated in the pilot phase prior to the onset of the study, 36 participated in the preintervention, and only 17 participated in both the pre- and post-intervention phases. It is descriptive data from these 17 nurses that we draw on here. Of the 17 nurses with complete data, most (82%) had two or more years of academic training; 53% had up to five years of nursing experience, 23.5% had 6–16 years of experience, and the remaining 23.5% had over 17 years of nursing experience.

#### 2.1.2. Caregivers and Children

 One hundred and seven caregiver-child pairs participated in the evaluation of the intervention (47% female babies, 53% males). Of the caregivers, 98% were the children's biological mother, while the remaining 2% were the children's grandmother. Upon admission to hospital, the majority of children were diagnosed with gastroenteritis (93%), while others were admitted for pneumonia (3%), bronchitis (1%), and other conditions (3%). Hospital stays ranged from 1 to 25 days (Mean = 6.25, SD = 5.14).

Demographic information was obtained from caregivers upon admission to hospital. The majority were single (77%), 16% reported cohabiting with a partner, 5% were married, and 2% were separated. The majority of caregivers lived with their parents (70%), 18% either rented or shared a home with others, and 12% lived in their own house.

### 2.2. Study Design

**Figure 1:**
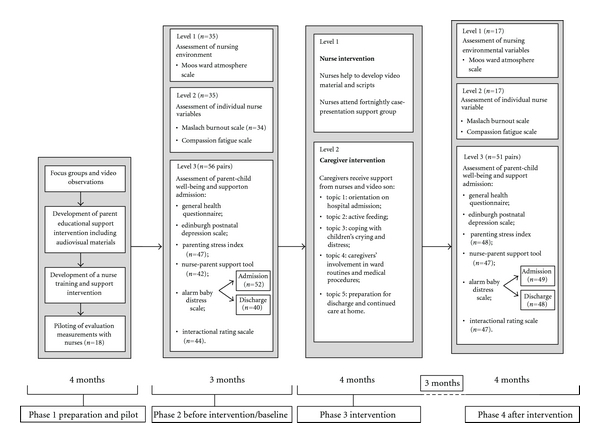
Overview of intervention design.

 The study included four major phases. A pilot phase preceded the first period of preintervention phase followed by the intervention phase, and then postintervention one. Child data were collected across the pre- and post-intervention phases within a nested individual case design. Specifically, child measures were assessed at admission and again upon discharge within both the pre- and post-intervention phases. See Figure 1 for an overview of the study design.

### 2.3. Measures

#### 2.3.1. Nurse Measures

The internal consistency of the measures used amongst the participants in the study was assessed by Cronbach's alpha.

**Compassion Fatigue Scale-Revised (CF-R)**

This 30-item modified version of the self test was designed to identify symptoms of compassion fatigue [16]. The total compassion fatigue score is derived from two scales: posttraumatic and/or secondary traumatic stress and burnout (Cronbach's alpha =  .92).

**Moos Ward Atmosphere Scale (WAS)**

This 40-item questionnaire was developed to determine staff and client perceptions of ward environment [17]. The three dimensions derived are: relationship, personal development, and system maintenance. (Cronbach's alpha =  .67).

**Maslach Burnout Inventory (MBI)**

The MBI is a 22-item questionnaire which assesses three aspects of nurse burnout: emotional exhaustion, depersonalization, and reduced personal accomplishment [18]. The current study employed the Human Services Survey (HSS) version, which was developed specifically to measure burnout in health care staff (Cronbach's alpha =  .85).

#### 2.3.2. Caregiver Measures

**Parenting Stress Index (PSI)**

The short form 36-item version was used in order to identify stressful areas in parent-child interactions [19]. The PSI yields a total stress score from three scales: parental distress, parent-child dysfunctional interaction, and difficult child (Cronbach's alpha =  .72).

**Nurse-Parent Support Tool (NPST)**

This 21-item scale measures caregivers' perception of nursing support received during their child's hospitalization [20]. This tool captures four overlapping aspects of nursing support: supportive communication and information giving, emotional support, parental esteem support, and instrumental support (Cronbach's alpha =  .89).

**General Health Questionnaire (GHQ)**

This 12-item psychological screening was developed to identify minor psychiatric disorder in caregivers [21] (Cronbach's alpha =  .69).

**Edinburgh Postnatal Depression Scale (EPDS)**

This 10-item questionnaire was used to detect mothers who may be suffering from depression [22] (Cronbach's alpha =  .72).

#### 2.3.3. Child Measures

**Alarm Distress Baby Scale (ADBB)**

This modified 5-item observer-rated scale assesses social withdrawal among infants [23]. The five scales included infants' facial expression, eye contact, and general level of activity, vocalizations, and ability to engage in a relationship with someone other than his/her caregiver. Two raters were trained using the manuals and jointly made assessments until full agreement was reached.

#### 2.3.4. Caregiver-Child Interaction Measure

**Interaction Rating Scale (IRS)**

Observers rated caregiver-child interactions on a 31-item interaction scale [24]. Observations of caregivers and children were made during two types of interactions: caregiver-child face to face interactions and feeding sessions. Again, agreement between two raters was established.

The study was approved by the Biomedical Research Ethics Committee at the Nelson Mandela School of Medicine, University of KwaZulu-Natal, and all participants provided written informed consent.

## 3. Results

### 3.1. Demographic Variables

 The various demographic data collected were compared between caregivers and children in the preintervention phase and those in the post-intervention phase. Type of caregiver income differed across the two groups, such that caregivers in the preintervention phase tended to have a single source of income (fixed income or government grant), while those in the postintervention phase reported having multiple sources of income. No social significance could be attached to the difference. Caregivers in the pre- and post-intervention phases did not differ on any other demographic descriptors. In addition, neither child age nor child gender differed across the two phases. See Table 1 for demographic information of caregivers and children.

### 3.2. Qualitative Description of Nurse Measures

 During the pilot study, nursing staff reported some compassion fatigue as well as low patient involvement in the ward and low levels of staff support for patients; nonetheless, they endorsed high levels of both personal development and system maintenance within the ward. With regards to burnout, nurses in the pilot study experienced high levels of emotional exhaustion, low levels of depersonalization, and average levels of personal accomplishment during the pilot phase of the study. The nurses enjoyed their active involvement in developing the interventions and found the interventions themselves empowering and relatable, as many were taken from video observations of their own nursing behaviors or of nurses they knew. For example, a tender and reassuring way in which a nurse held eye contact with a baby during a blood draw which seemed to have a calming effect on the child; how a nurse helped a child to eat their food, or the manner in which they explained to a mother the value of leaving a comfort object for the child to hold in their absence. However, as reported by Zuma [15], there were no significant changes in nurses' compassion fatigue, perceptions of ward environment, and levels of burnout across the pre- and post-intervention phases.

### 3.3. Quantitative Analysis of Caregiver Measures

Table 2 provides descriptive data for the various caregiver measures. An analysis of variance (ANOVA) was performed to determine whether caregivers in the pre- and post-intervention phases differed with respect to the various measures. The results revealed no significant differences between the two groups on the GHQ, EPDS, or PSI. Caregivers in the two phases, however, did report different levels of nursing support on the NPST (*F*(1, 87) = 27.96, *P* < .001). Specifically, caregivers in the postintervention group reported receiving significantly more support from nursing staff than caregivers in the preintervention group. To test the possibility that duration of hospital stay may have had an impact on caregiver outcomes, a multivariate analysis of variance (MANOVA) was conducted. The main effect of study phase remained significant (Wilks' *λ* = .59, *F*(6,46) = 5.45, *P* < .001) with caregivers in the postintervention phase reporting receiving significantly higher levels of nursing support than caregivers in the preintervention phase, and there was neither a main effect of duration of hospital stay (Wilks' *λ* = .15, *F*(96,269.43) = 1.11, *ns*), nor an interaction between intervention phase and duration of hospital stay (Wilks' *λ* = .34, *F*(54,239.15) = 1.05, *ns*). This suggests that the amount of time caregivers and children spent in hospital did not have a significant impact on the various caregiver measures across the two study phases.

The main improvements mentioned by mothers concerned nurse communication and friendliness, and the willingness of nurses to provide caregivers with comfort, respond to their concerns, and encourage them to be involved in their children's care. For example mothers noted:

*“Nurses helped me by being open, I was able to talk to them whenever I wanted.”*

*“Most nurses were able to make jokes - they had a smile that made me feel I could talk to them with whatever I needed”*

*“Nurses helped me by passing on my concerns to doctors and they talked to me about general things not related to the baby.”*

### 3.4. Quantitative Analysis of Child Measures

 Paired *t*-tests were conducted to compare infant social withdrawal behaviour assessed by the alarm distress baby scale (ADBS) on admission and discharge in both the pre- and post-intervention conditions. Infants did not differ between admission and discharge in the preintervention phase (*t*(39) = .81, *ns*). Infant social withdrawal behaviour was, however, significantly reduced between admission and discharge during the postintervention phase (*t*(47) = 6.53, *P* < .001). See Table 3 for descriptive data on the alarm distress baby scale.

Table 4 provides descriptive statistics for the various interaction rating scale ratings. An ANOVA was performed to assess differences in caregivers' and children's interactions with one another across the pre- and post-intervention phases using Field's interaction rating scale. There were no differences between the pre- and post-intervention phases in caregivers' behaviours during both face-to-face interactions and feeding sessions. Children in the two groups also did not differ in the face-to-face interactions; however, for the feeding sessions, children in the intervention group were rated as being significantly more alert, more relaxed, and more engaged with caregivers than those children in the baseline group, *F*(1, 105) = 8.73, *P* < .01. For instance, during feeding sessions, babies in the postintervention phase were rated by observers as having a “relaxed body and molding to mother,” displaying “rare head aversion,” and “frequently looks at mother.” An analysis of variance (ANOVA) was conducted to examine the effect of duration of hospital stay on children's feeding behavior across pre- and post-intervention. Results indicated that there was neither a significant main effect of study phase (*F*(1,46) = 1.61*, ns*) nor of duration of hospital stay (*F*(16, 46) = .52, *ns*). In addition, the interaction was also nonsignificant (*F*(10, 46) = 1.41, *ns*), suggesting that length of stay in hospital did not have a significant impact on children's feeding behavior across the two study phases.

## 4. Discussion and Conclusion

Our evaluation of the intervention revealed differences in three areas indirectly and directly linked to the social emotional care of children during their stay in hospital and their subsequent recovery. The first is that mothers rated nurses as more supportive after the intervention, suggesting that the intervention had resulted in medium-term changes in the way nurses addressed and interacted with caregivers in the ward. As emphasized in the extant literature, there is a great need to involve parents and families in the care of hospitalized children [25]. Our results indicate that by building a stronger partnership with nurses through increased support and guidance, caregivers feel more empowered and confident in the care of their children during a potentially difficult and stressful hospitalization. The second is in rated feeding behavior, with children being observed as more relaxed, alert and engaged during feeding in the postintervention phase. During the baseline we noted high levels of anxiety experienced by mothers during feeding, as they were exhorted to ensure that their children receive nourishment. The intervention specifically addressed the need for responsiveness and calm determination in feeding a young sick child with a poor appetite and potentially sores in their mouth. By demonstrating that simple techniques employed during the intervention can significantly ameliorate children's feeding experience during their hospital stay, we can better ensure children's nutrition and the likelihood of their recovery to health. Finally, babies were rated as less socially withdrawn on discharge as compared to admission during the post- as compared to the pre-intervention phase. It is possible that by engaging mothers and giving them confidence through increased knowledge and support from nurses, and by improving children's feeding behaviours, children received the necessary stimulation and care required to improve their health, as reflected in the significant decreases in children's social withdrawal.

Despite the significant findings of the evaluation, the study also illustrates the complexity of undertaking real-world evaluations of interventions conducted under changing conditions. Given the important lessons that can be gained for future research, we outline some of the challenges here. Some challenges include nurses' rotations on and off the ward through variable staff schedules; caregivers being limited to intermittent hospital visits as their finances, work and home responsibilities allow; and children being admitted for varying lengths of stay with widely differing medical conditions and prognoses. A small number of children died between admission and discharge in both the pre- and post-intervention phases. There is also the challenge of using measures developed in the west for very different social, cultural, and economic conditions. Nonetheless, measurement consistency was acceptable and demonstrated the robustness of constructs such as burnout and compassion fatigue, as well as being sensitive in areas to which the intervention was directed, such as nurse support for caregivers, responsive feeding, and techniques for calming young children when they are distressed.

The pattern of the results manifests some of the limitations of the intervention itself. The intervention did not address structural difficulties in the administration of the hospital, such as the rotation of nurses, not only between night and day shifts, but between hospital wards with varying tasks in different areas of nursing care. Many nurses, when on the paediatric ward, expressed a preference to stay in one or other particular area of nursing rather than rotate through all sections of a general hospital. Lack of change in working conditions and administration was reflected in the absence of differences between pre- and post-measures on assessments of the ward atmosphere.

Similarly the intervention did not address the many difficulties faced by caregivers. The majority of mothers learned their HIV status for the first time when their sick child was admitted to hospital and tests for HIV, leading to an assessment of their own health status [26–28]. Demoralisation and depression are common [29, 30] in addition to socioeconomic pressures, anxiety about a sick child, challenges of care for other children left at home, and the difficulties of getting off work from generally part-time or insecure jobs to spend time with a young child in hospital. The intervention was directed at increasing nurse empathy for caregivers and even increasing interaction between mothers so that it was not uncommon to see a mother who was visiting her child get up to attend to another child on their own when they were distressed. But more work is needed to address the broader context of women's lives and the stresses of their own and their family circumstances, made considerably worse by the seriousness of the illness experienced by their child and their general powerlessness to remedy their child's condition [31] as well as the knowledge of their own HIV status with its attendant self- and other-stigmatization [32, 33]. The lack of change in these areas of women's lives is reflected in the absence of differences between pre- and post-test assessments of caregiver emotional state and well-being through the General Health Questionnaire, the Edinburgh Postnatal Depression Scale, and the Parenting Stress Index.

In conclusion, evaluation of interventions of these types, in these challenging circumstances, is important. Resources are limited and decisions to improve the care of children must be based on the best possible evidence. Despite the challenges of the evaluation, the measures show promise as does the intervention, limited in its scope to nurse-caregiver-child interactions, as it was. Our evaluation provides evidence that simple, cost-effective interventions can be implemented in a child health context not only to directly improve the health and social care of children, but also indirectly by involving parents and families in delivering nursing care through the promotion of a partnership between nursing staff and caregivers. Changes in ward and hospital administration are also needed, as are outreach support services for families affected by HIV and AIDS, but both were beyond the scope of the project. Nonetheless, the success of the project as perceived by hospital staff and health officials has led to a second phase inwhich we have developed a training and support programme for home-based palliative care for young children supported by the Diana Princess of Wales Memorial Fund. With greater support at home, young children living with HIV and their families may be able to avoid unnecessary and costly hospital admissions for conditions that can be managed with home-based nursing and palliative care.

## Figures and Tables

**Table 1:** Caregiver demographic information by study phase.

Variable	Before intervention	After intervention
	*N * (%)	*N * (%)
Child age (months)		
0.25–4	18 (32.1%)	20 (39.2%)
5–8	16 (28.6%)	15 (29.4%)
9–12	11 (19.6%)	4 (7.8%)
13–16	4 (7.1%)	6 (11.8)
17–60	7 (12.5%)	6 (11.8)
Caregiver age (years)		
15–20	10 (17.8%)	9 (17.6%)
21–25	16 (28.6%)	19 (37.3%)
26–30	16 (28.6%)	9 (17.6%)
31–35	7 (12.5%)	9 (17.6%)
35–51	7 (12.5%)	5 (0.9%)
Marital status		
Single	45 (80.3%)	38 (74.5%)
Cohabiting	6 (10.7%)	11 (21.6%)
Married	3 (5.4%)	2 (3.9%)
Widowed	2 (3.6%)	0 (0.0%)
Help with childcare		
Yes	43 (76.8%)	45 (88.2%)
No	13 (23.2%)	5 (9.8%)
Education		
No education	0 (0.0%)	0 (0.0%)
Schooling	54 (96.4%)	50 (98%)
After school	2 (3.6%)	1 (2%)
Income		
Fixed income	19 (33.9%)	3 (5.9%)
Social security grant	8 (14.3%)	14 (27.4%))
Fixed income and grant	12 (21.4%)	0 (0.0%)
Grant and other	17 (30.3%)	34 (66.7%)
Housing		
Own house	9 (16.1%)	4 (7.8%)
Share house/rental	7 (12.5%)	12 (23.5%)
Family	40 (71.4%)	35 (68.6%)

**Table 2:** Caregiver measures by study phase.

Measure	Preintervention		Postintervention	
	M	SD	M	SD
General Health Questionnaire	8.00	2.22	8.61	2.49
Edinburgh Postnatal Depression Scale	17.38	4.57	17.92	5.09
PSI—parental distress subscale	40.33	6.25	41.60	6.10
PSI—parent-child dysfunctional				
Interaction subscale	61.85	8.94	64.15	7.88
PSI—difficult child subscale	33.48	5.63	33.88	3.99
PSI—total score	135.66	15.06	139.64	14.16
Nurse-parent support tool	3.10*	0.61	3.65*	0.46

*P < .001.

**Table 3:** Alarm distress baby scale.

	Preintervention		Postintervention	
	M	SD	M	SD
Admission	.27	.22	.61*	.49
Discharge	.21	.40	.08*	.23

*P < .001.

**Table 4:** Interaction rating scale by study phase.

Scales	Preintervention		Postintervention	
	M	SD	M	SD
Caregiver scales				
Face-to-face	2.50	0.18	2.50	0.24
Feeding	2.61	0.15	2.61	0.15
Child scales				
Face-to-face	2.53	0.19	2.48	0.22
Feeding	2.74*	0.24	2.88*	0.23

*P < .01.
